# Malignant germ cell tumours of childhood: new associations of genomic imbalance

**DOI:** 10.1038/sj.bjc.6603602

**Published:** 2007-02-06

**Authors:** R D Palmer, N A Foster, S L Vowler, I Roberts, C M Thornton, J P Hale, D T Schneider, J C Nicholson, N Coleman

**Affiliations:** 1 MRC Cancer Cell Unit, Hutchison/MRC Research Centre, Box 197, Hills Road, Cambridge, CB2 2XZ, UK; 2 Department of Paediatric Oncology, Addenbrooke's Hospital, Box 181, Hills Road, Cambridge, CB2 2QQ, UK; 3 Centre for Applied Medical Statistics, Department of Public Health & Primary Care, University of Cambridge Forvie Site, Robinson Way, Cambridge, CB2 2SR, UK; 4 Department of Pathology, Institute of Clinical Science, Royal Group of Hospitals, Grosvenor Road, Belfast, BT12 6BA, UK; 5 Department of Paediatric Oncology, Sir James Spence Institute of Child Health, Royal Victoria Infirmary, Queen Victoria Road, Newcastle Upon Tyne, NE1 4LP, UK; 6 Department of Pediatric Oncology, Heinrich Heine Universität, Moorenstraße 5, 40225, Düsseldorf, Germany

**Keywords:** neoplasms, germ cell, child, gonadal, extragonadal, comparative genomic hybridisation

## Abstract

Malignant germ cell tumours (MGCTs) of childhood are a rare group of neoplasms that comprise many histological subtypes and arise at numerous different sites. Genomic imbalances have been described in these tumours but, largely because of the paucity of cases reported in the literature, it is unclear how they relate to abnormalities in adult MGCTs and impact on potential systems for classifying GCTs. We have used metaphase-based comparative genomic hybridisation to analyse the largest series of paediatric MGCTs reported to date, representing 34 primary tumours (22 yolk sac tumours (YSTs), 11 germinomatous tumours and one metastatic embryonal carcinoma) occurring in children from birth to age 16, including 17 ovarian MGCTs. The large dataset enabled us to undertake statistical analysis, with the aim of identifying associations worthy of further investigation between patterns of genomic imbalance and clinicopathological parameters. The YSTs showed an increased frequency of 1p- (*P*=0.003), 3p+ (*P*=0.02), 4q− (*P*=0.07) and 6q− (*P*=0.004) compared to germinomatous tumours. Gain of 12p, which is invariably seen in adult MGCTs, was present in 53% of primary MGCTs of children aged 5–16 and was also observed in four of 14 YSTs affecting children less than 5. Two of these cases (14% of MGCTs in children less than 5) showed gain of the 12p11 locus considered to be particularly relevant in adult MGCTs. Gain of 12p showed a significant association with gain of 12q. Conversely, MGCTs without 12p gain displayed a significantly increased frequency of loss on 16p (*P*=0.04), suggesting that this imbalance may contribute to tumour development in such cases. This data provides new insight into the biology of this under-investigated tumour group and will direct future studies on the significance of specific genetic abnormalities.

Germ cell tumours (GCTs) are a complex group of heterogenous tumours that comprise both benign and malignant histologies. Children in the UK under 16 years of age are treated on paediatric childhood cancer treatment schedules, overseen by Children's Cancer and Leukaemia Group (CCLG). Teratomas in children, whether mature or immature, are deemed benign and treated by surgery alone (Palmer *et al*, 2003). Chemotherapy is reserved for malignant histologies and the rare occurrence of immature teratoma with widespread seeding (typically peritoneal), when it is used in an attempt to reduce further spread and facilitate surgery. These treatment schedules are different from those used in adults, suggesting that GCTs of adult and childhood are likely to differ biologically.

We are investigating the biology of paediatric malignant germ cell tumours (MGCTs), a diverse group presumed to have a common cell of origin, the primordial germ cell. These tumours occur at several different anatomical sites and, unlike their adult counterparts, only 50% occur in the gonad. The remainder arise in various midline or near-midline sites, presumed to be due to arrested or aberrant migration of the primordial germ cells during foetal development. There is a bimodal age distribution of childhood MGCTs. The MGCT seen in infancy is the yolk sac tumour (YST), which predominantly arises in the testis but does also occur in alternative sites, for example within sacrococcygeal teratomas (SCT). By later childhood, adolescence and early adulthood, MGCTs are seen in a range of sites, including the ovary, brain and mediastinum, and may be of various histological types, especially embryonal carcinoma (EC), choriocarcinoma, YST and germinomatous tumours (including testicular seminoma and ovarian dysgerminoma).

Particular histologies are reported to show similar patterns of genomic imbalance irrespective of the site in which they occur (Riopel *et al*, 1998; Perlman *et al*, 2000; Rickert *et al*, 2000; Schneider *et al*, 2002; Schneider *et al*, 2006), consistent with the hypothesis that they arise from the same progenitor cell. Recently, a new classification system for GCTs was proposed (Oosterhuis and Looijenga, 2005), dividing GCTs into five types. In this classification, early childhood tumours (typically under 5 years of age and almost exclusively teratoma or YST) are referred to as type I tumours, and separated from the remainder of MGCTs that occur in the rest of childhood or throughout the majority of adulthood. Type II tumours represent all histologies except benign ovarian teratoma (type IV), spermatocytic seminoma (type III) and hydatidiform moles (type V). This classification was based upon the epidemiological and biological differences so far determined between GCT histologies. An important factor in this regard is said to be the lack in type I tumours of 12p gain (other than gain of small, predominantly telomeric regions in some cases). In contrast, gain of 12p is reported to occur in all type II MGCTs (Oosterhuis and Looijenga, 2005).

Genomic copy number imbalances (CNIs) in MGCTs of adulthood have been extensively investigated. Gain of 12p, which is generated by formation of an isochromosome [iso(12p)] in up to 80% of cases of adult testicular GCTs (TGCTs), appears to be a specific and early cytogenetic event in all histologies in adults (Korn *et al*, 1996; Kraggerud *et al*, 2000; Looijenga *et al*, 2000). Additional karyotypic abnormalities in these tumours include gains on 1, 7, 8, 12, 21, and X, as well as losses on 11, 13 and 18 (Summersgill *et al*, 1998; van Echten *et al*, 2002), but none is found as consistently as 12p gain. In contrast, genomic CNIs in childhood MGCTs, whether deemed type I or II, have received comparatively little investigation. There have been several investigations of small numbers of cases, generally as a component of studies that also included teratomas. A wide range of CNIs has been described in childhood MGCTs, including gains on 1q, 2p, 3, 7, 8, 13, 14, 20q, 21, and X, as well as losses on 1p36, 4q, 6q, 11, 13 and 18; but again none is seen consistently (Mostert *et al*, 2000; Perlman *et al*, 2000; Schneider *et al*, 2002; van Echten *et al*, 2002; Schneider *et al*, 2006). In all these studies, comparative genomic hybridisation (CGH) findings were consistent with previous cytogenetic data and with verification studies based on fluorescent *in situ* hybridisation. Metaphase chromosomes have generally been used for CGH and only limited additional information appears to have been gained in the analysis of childhood MGCTs by using 1 MB CGH arrays (Veltman *et al*, 2005).

An important reason why it is not possible to identify with confidence which CNIs are important in childhood MGCTs is that data from only 82 samples (where age and CGH profile are presented) of these heterogenous tumours have been reported to date. This lack of information has clinical implications. For example, there is uncertainty concerning whether MGCTs in adolescents should be regarded as being of adult or childhood type and which algorithms are required to inform this decision. We reasoned that a single large study would allow investigation of CNIs in childhood MGCTs in relation to histology and clinicopathological data and enable statistical investigation of associations between CNIs.

We have therefore used metaphase CGH to identify CNIs worthy of further investigation in childhood MGCTs. We deliberately chose cases from patients of 16 and under, in whom paediatric treatment schedules are applied in the UK. We used a large series of 34 paediatric MGCTs, representing the entire Children's Cancer and Leukaemia Group (CCLG) bank of frozen tumours available at the time of the study, together with additional unselected cases made available from European collaboration with Heinrich Heine University, Düsseldorf. This study therefore represents the most comprehensive genomic analysis of this tumour group reported to date. We have identified previously unreported CNI associations of potential significance in the biology of childhood MGCTs and, in particular, have observed that gain of 12p (including 12p11) is reasonably common in YSTs of children under 5.

## MATERIALS AND METHODS

### Tumour samples

Banked frozen tissue was provided by the CCLG from children 16 years of age or younger. A total of 29 tumours from United Kingdom were treated on the extracranial GC 8901 (1989–2004) study, with three additional specimens made available from the CCLG intracranial germ cell tumour study and two unselected specimens from German GPOH-MAKEI studies. Following Multi-centre Research Ethics Committee approval (ref 02/4/071), tumour material was transported to Cambridge from the banking centres across the UK and Germany for analysis. In total, 34 tumours were analysed, each from a different patient. None of the tumours had been included in previous studies. Thirty-three cases were primary tumours, while one was a pulmonary metastasis of an EC of the testis. Nineteen tumours were stage 1, six stage 2, two stage 3 and seven stage 4. Two children died (one of disease, one unrelated) and five relapsed (three of whom had stage 1 disease and received no primary chemotherapy), with follow-up ranging from 12 to 144 months in disease-free cases.

Each case was provided with a histological diagnosis following CCLG expert panel review. Patient characteristics are shown in Table 1. All specimens examined in the study initially underwent frozen section analysis, with independent review by three separate histopathologists, two of whom are consultant paediatric histopathologists. This process enabled confirmation of the histopathological diagnosis and the presence of at least 90% tumour cells in each frozen tissue sample.

### DNA isolation

Sections of tumour were homogenised (PolyTron PT2100) in TRIzol (Invitrogen, Paisley, UK) to preserve nucleic acids. Following the addition of chloroform, DNA was extracted from the interphase/organic phase using ethanol precipitation. The resultant DNA pellets were serially washed with 0.1 M sodium citrate in 10% ethanol, before resuspension in water (pH 8.4).

### CGH microscopy and analysis

CGH was performed as described previously (Alazawi *et al*, 2004) but with modifications. Briefly, 50 ng of tumour DNA and male peripheral blood leucocyte reference DNA were amplified and labelled by two-step degenerate oligonucleotide-primed (DOP) PCR using 6 MW primer (Foster *et al*, 2005). Test DNA was labelled with biotin-16-dUTP and reference DNA with digoxigenin-11-dUTP (Roche, Lewes, UK). DOP-PCR products were combined with 5 *μ*g Cot DNA (Roche, Lewes, UK) to form probes before hybridisation with normal male metaphase spreads (Vysis, Richmond, UK) at 42°C for 48 h. Following stringency washes, detection was performed with streptavidin-Cy3 antibody (Amersham Biosciences, Little Chalfont, UK) and anti-digoxigenin-FITC antibody (Roche, Lewes, UK). Chromosomes were counterstained with DAPI and images captured using an Axioplan II fluorescence microscope equipped with filter sets for DAPI, FITC and Cy3 (Chroma, Rockingham, VT, USA). Images were acquired using SmartCapture 2001 software (Digital Scientific, Cambridge, UK) and analysed using Quips CGH karyotyper (Vysis, Downers Grove, IL, USA).

At least 10 metaphases were analysed for each sample. The thresholds used for detection of loss and gain were 0.8 and 1.2, respectively, based on normal:normal hybridisations using normal gonadal tissue (data not shown). As the same normal male control DNA was used to analyse tumours of male and female patients, only autosomal CNIs were assessed.

### Statistical analysis

Analysis was carried out in SPSS V13.0. (SPSS Inc, Chicago, IL, USA) and StatXact V4.0 (Cytel, Cambridge, MA, USA). For each chromosome arm, gains were compared to nongains (i.e. ‘no change’ or ‘loss’) and losses were compared to nonlosses (i.e. ‘no change’ or ‘gain’) using the confidence interval on the difference of two proportions. These proportions were compared between different attributes of the dataset, for example YST *vs* germinoma, males *vs* females, etc. The overall aim of the data analysis was to identify CNI associations worthy of further investigation. In view of this, although multiple tests were performed, associations were defined as significant where the *P*-value was less than or equal to 0.05.

## RESULTS

### Characteristics of childhood MGCTs analysed

We examined 34 MGCTs from 34 different patients (22 female, 12 male). Of these, 33 cases were primary tumours and one was a metastasis (Table 1). There were 11 germinomas (eight dysgerminomas, two intracranial germinomas and one arising within an ovarian teratoma), 22 YSTs (eight ovarian, seven testicular, five SCT, one intracranial, one vaginal) and one EC (pulmonary metastasis from a testicular primary). Twenty-four of the primary tumours were gonadal (17 ovarian, seven testicular) and nine were extragonadal. There was one potential case of gonadal dysgenesis (case 24), where the contralateral gonad was considered to be a streak ovary intraoperatively. Otherwise no tumour included in this study was from patients diagnosed with gonadal dysgenesis (as determined by the intraoperative findings and long-term clinical follow-up). Fourteen MGCTs occurred in children less than 5 years old at diagnosis. All of these were YSTs, of which seven were testicular, five arose within a SCT, one was ovarian and one vaginal. Twenty MGCTs occurred in children 5 years or older, of which 16 were ovarian, three intracranial and one testicular. These samples included all 11 germinoma cases. The clinicopathological features of the cases analysed were largely representative of childhood MGCTs in general (Palmer *et al*, 2003; Schneider *et al*, 2004), other than a relative preponderance of ovarian samples. Seven tumours represented a single MGCT component within a teratoma and the samples analysed from these cases were completely or predominantly (>90%) composed of the malignant element (Table 1).

### CNIs in the entire sample set

**Figure 1:**
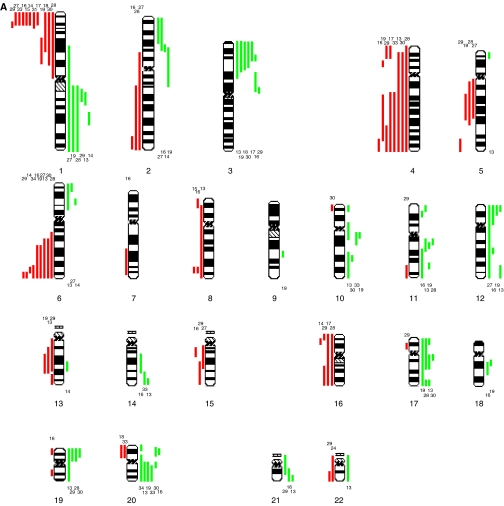

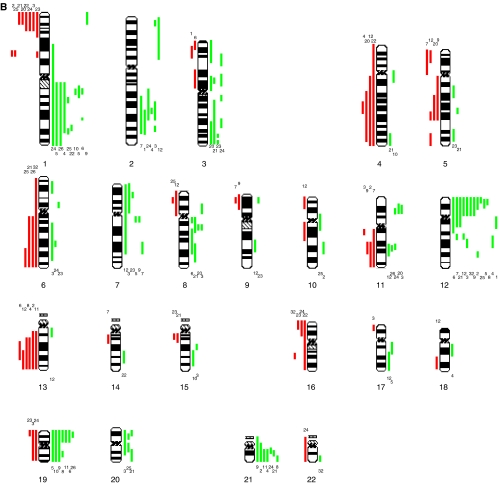
Summary karyograms for all 34 MGCTs analysed. Gains are shown by green bars to the right of each chromosome ideogram and losses as red bars to the left of each ideogram. Only autosomes are displayed. The numbers above or below the bars refer to individual samples. (**A**) Summary karyogram for all 14 MGCTs in children <5 years of age. (**B**) Summary karyogram for all 20 MGCTs in children aged ⩾5 & <16.

The number of chromosomes showing CNIs varied from 1 to 24 per tumour (mean 9), with slightly more CNIs per YST (mean 9.5, range 1–24) than per germinoma (mean 7.1, range 2–18) (Figure 1), although the difference was not significant (*P*=0.32, Mann–Whitney *U* test). The EC showed 17 CNIs. The numbers of CNIs are equivalent to those in previous studies (Summersgill *et al*, 1998; Kraggerud *et al*, 2000; Mostert *et al*, 2000; Schneider *et al*, 2002; Kildal *et al*, 2004). Commonly observed CNIs in the entire sample set (i.e. irrespective of histology) included gains on 1q (44%), 12p (44%), 12q (32%), 19p (32%), 20q (26%), 21 (29%) and losses on 13 (26%). The most prevalent abnormality seen by us was loss on 1p, which was detected in 20 (59%) of all MGCTs and 18 (82%) of YSTs. Less frequently, gains on 2q, 6p, 10q, 11p and 20p were observed (Figure 1).

### YSTs *vs* germinomas

**Figure 2:**
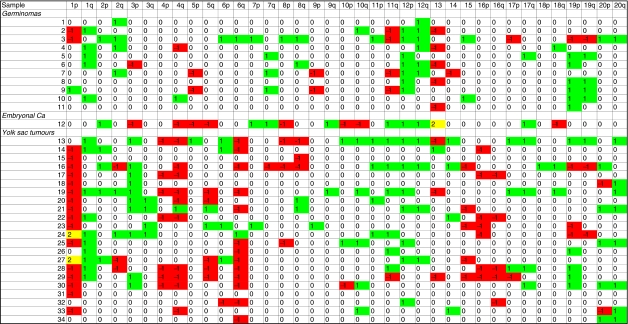
Summary of detectable CNIs on each chromosome arm for all 34 paediatric MGCTs analysed. Gains=green, scored 1; losses=red, scored −1; both gain and loss=yellow, scored 2.

YSTs were significantly more likely than germinomas to contain gains on 3p and losses on 1p and 6q, with a borderline significant increase in the frequency of loss on 4q (*P*=0.07) (Figure 2). These imbalances were more typically seen in YSTs of children less than 5 (in whom the tumours were testicular) than in YSTs of children greater than 5 (in whom the tumours were typically ovarian). Germinomas showed borderline significant increases in the frequency of gain on 12q (*P*=0.07) and 19q (*P*=0.095), and loss on 11q (*P*=0.095) (Figure 2).

### Tumour site, stage and patient age

Of the nine extragonadal primary MGCTs analysed, three were intracranial, five sacrococcygeal (SCT) and one vaginal. The CNIs seen in these cases were equivalent to those in gonadal tumours of similar histologies, consistent with the notion that these tumours are likely to have arisen from the same cell of origin, albeit aberrantly sited in the extragonadal cases. Testicular MGCTs, regardless of type, were more likely than ovarian MGCTs to have gain on 3p and loss on 8q. A greater number of CNIs per tumour was observed with increasing stage (mean CNIs of 9.2 for stage 1 and 11.7 for stage 4), although there was no evidence of an association between stage and any particular CNI. MGCTs occurring in children less than 5 years of age were more likely to contain loss on 1p, 4p, 4q and 6q compared to MGCTs in children of 5 years or more (Table 2).

### Chromosome 12p status

**Figure 3:**
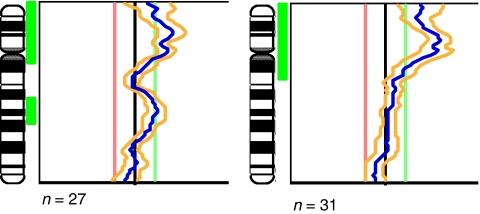
Summary fluorescent ratio profiles for chromosome 12 in two gonadal YSTs, showing the mean fluorescent ratio profile (blue line) and 95% confidence intervals (yellow lines). The left-hand image is from a pure YST of the testis in a 4-year-old (sample 16); and the right-hand image is from a pure YST of the ovary in a 13-year-old (sample 21).

Gain on chromosome 12p, which is invariably found in adult MGCTs (Korn *et al*, 1996), was observed in 15 of the 34 (44%) MGCTs analysed (seven germinomas, seven YSTs and one EC). 12p gain was more prevalent in the germinomatous tumours than the YSTs, but not significantly so (64 *vs* 32%; *P*=0.11). 12p gain was present in four of the 14 YSTs that occurred in children less than 5 years old. The regions of gain in these cases were whole chromosome 12 (case 27), 12p11-pter (case 16), 12p12-pter (case 34) and 12p13-pter (case 13) (Figure 3). Two of the 14 cases therefore showed gain at the 12p11 locus typically gained in adult MGCTs. Gain of 12p was more common in children 5 or over, yet occurred in only 10 of the 19 (53%) primary MGCTs in this age group. There was no evidence of a significant difference in the frequency of 12p gain in primary MGCTs of children aged 5 or over compared to MGCTs of children under 5 (53 *vs* 29%; *P*=0.19).

When analysing all cases combined, gain on 12p showed a significant association with gain of 12q (*P*=0.01), but there was no evidence of a significant association with any other CNI (Table 2). When considering only the MGCTs showing 12p gain, tumours in children less than 5 were significantly more likely to show gain on 2p, 3p, 11q and loss on 6q than tumours in children of 5 years or more (Table 2). In the YSTs (all ages), tumours showing 12p gain were significantly more likely to show loss of 6q and gain of 12q compared to tumours without 12p gain (Table 2).

Tumours in which 12p gain was absent were more likely to show 16p loss than samples in which 12p gain was present. This applied to all cases combined and also to the YSTs alone (*P*=0.04 for both) (Table 2). The smallest region of overlap on 16p in all cases combined was at 16p12–13.1 (Figure 1).

## DISCUSSION

The 34 childhood MGCTs analysed in this study constitute the largest series so far reported and represents a substantial addition to the 82 cases published to date (see Table 3, which includes data from the present study, but excludes published cases without a CGH profile, cases reported on more than one occasion and cases where the age of the patient was not stated). We have confirmed the existence of previously recognised CNIs in MGCTs of childhood, such as losses on 1p, 4, 6q and 13, as well as gains on 1q, 12p, 20 and 21 (Perlman *et al*, 2000; Oosterhuis and Looijenga, 2005; Veltman *et al*, 2005). In addition, the relatively large dataset in our study has enabled us to undertake statistical investigations and thereby identify novel associations that may be relevant in the pathogenesis of childhood MGCTs.

It has previously been reported that gain of 12p is invariably found in adult TGCTs (Korn *et al*, 1996), typically as a result of iso(12p) formation. Similarly, 12p gain is exceedingly common in adult ovarian MGCTs (Riopel *et al*, 1998; Kraggerud *et al*, 2000). In tumours at both sites gain of 12p11–12 (i.e. proximal 12p) is of particular importance and there is evidence for a potential role for genes in this region in the suppression of apoptosis (Zafarana *et al*, 2002). In contrast, gain of 12p has been inconsistently reported in childhood MGCTs, with gain of 12p13-pter observed more frequently than gain of more proximal 12p (Oosterhuis and Looijenga, 2005). This has therefore formed one of the discriminatory cytogenetic features between adult and paediatric (particularly infantile) MGCTs. Interestingly, there is some evidence that 12p gain (whether 12p11 or whole arm) is less frequent in post-pubertal childhood MGCTs than in adult MGCTs (Rickert *et al*, 2000; Schneider *et al*, 2002) (Table 3), suggesting that adolescent tumours show biological heterogeneity, which is greater than in adult MGCTs.

It was reported by Veltman *et al* (2005) that gain of the whole 12p arm or iso(12p) has not been described in GCTs of neonates or children under 5 years old. In our series there were four cases with gain of 12p in children under 5, representing gains of whole chromosome 12 (case 27), 12p11-pter (case 16), 12p12-pter (case 34) and 12p13-pter (case 13). Furthermore, literature review indicates that 12p gain (not including isolated 12p13-pter gain) has been reported in approximately 15% of MGCTs occurring in children less than 5 years of age, whether analysed by CGH (Table 3), or kayotyping &/or *in situ* hybridisation (Table 4).

It therefore appears that while 12p gain is less frequent in childhood MGCTs than in their adult counterparts, and less frequent in the very young than in older children, the abnormality can occur in even very young children, where it may well be of biological and clinical importance. It has been reported that 12p11.2–12.1 gain is associated with progression from carcinoma *in situ* to malignancy in adult TGCTs (Ottesen *et al*, 2003) and that a 12p amplicon results in more rapid progression in this setting (Looijenga *et al*, 1999). As the literature describes so few paediatric MGCTs, it is not known whether gain of whole arm 12p, or isolated 12p11–12.1, will confer any prognostic disadvantage in children with MGCTs; especially where treatment uses less toxic, carboplatin-based chemotherapy schedules. However, it is interesting that 12p gain was seen in both of the childhood MGCT relapses reported to date (Riopel *et al*, 1998; Rickert *et al*, 2000). In a complimentary manner, the typical cytogenetic changes of YSTs in infants (loss on 1p, 4q and 6q) were also seen in YSTs of children over 5 years (always ovarian in this study), but with a reduced frequency. This is in keeping with published findings in pure histology YSTs affecting adolescents and adults, irrespective of tumour site (Speleman *et al*, 1990; de Bruin *et al*, 1994; Korn *et al*, 1996; Mostert *et al*, 1996; van Echten *et al*, 1996; Riopel *et al*, 1998; Summersgill *et al*, 1998; Kraggerud *et al*, 2000; Schneider *et al*, 2006). These findings suggest that whereas biological differences may exist between MGCTs of similar histology, distinction based simply on chronological age may not be appropriate.

We investigated which other CNIs may be important in the presence or absence of 12p gain in paediatric MGCTs. When analysing all cases combined, 12p gain was significantly associated with gain of 12q (*P*=0.01). As gain on 12p and 12q may represent aneusomy for chromosome 12, it would be interesting to investigate whether cases showing both CNIs have different clinicopathological associations (e.g. histology, patient age), compared to MGCTs showing 12p gain without 12q gain. However, this exercise would require a considerably larger sample size than was available for the present study. For the tumours showing 12p gain, those affecting children less than 5 years of age were more likely to show gain on 2p, 3p, 11q, and loss on 6q, than those affecting children of 5 or more (in which 12p gain may be of paramount importance). As all of the children under 5 had YSTs, this observation may reflect a feature of the tumour type (Table 2). Gain of 2p has been reported as a relatively infrequent event in MGCTs of childhood, notably YSTs (Rickert *et al*, 2000; Schneider *et al*, 2002), but the association with 12p gain may be particularly relevant.

In childhood MGCTs where 12p gain is absent, it is likely that other CNIs are of fundamental significance. Past work has largely focused on the possibility that tumour suppressor genes exist on 1p or 6q, especially in infantile YSTs (Perlman *et al*, 2000; Hu *et al*, 2001), although no candidate genes have yet been identified. In our study, we discovered that in the absence of 12p gain the most significant abnormality is loss of 16p (*P*=0.04), irrespective of histology. This raises the possibility that loss of one or more tumour suppressor genes on 16p, particularly at the smallest region of overlap of 16p12–13.1, may be an important event in these tumours. Since many statistical tests were carried out in our analysis, it will be important first to confirm the new associations that we have discovered in an independent sample set. Nevertheless, the identification in our study of abnormalities reported by other groups lends credence to the novel associations that we have identified.

Recently, a new classification system has been proposed that separates GCTs into five types (Oosterhuis and Looijenga, 2005), where the most important discrimination in paediatric cases is between type I and II tumours. The discrimination is essentially age related, with the transition at 4–5 years, since it is rare to have dysgerminoma below this age, or testicular tumours above this age until the onset of puberty. Cytogenetically, malignant type I tumours (typically testicular YSTs in boys under 5) are reported to show gain on 1q, 12p13 and 20q, as well as loss on 1p, 4 and 6q. Type II tumours (of any histological type other than mature teratoma) have gain on chromosome 7, 8, 12p, 21 and X, with loss of 1p, 11, 13 and 18 (Kraggerud *et al*, 2000; Rickert *et al*, 2000; Oosterhuis and Looijenga, 2005). Our study confirms that loss on 1p, 4 and 6q are strongly associated with MGCTs (invariably YSTs) of children under the age of 5 years (Table 2), and therefore putative type I tumours. We also observed an increase of borderline significance in the frequency of gain on 3p (*P*=0.06) in the tumours occurring in children under 5, which has been previously described in this age group in smaller studies (Mostert *et al*, 2000; Schneider *et al*, 2002; Veltman *et al*, 2005). However, we do not confirm that gain of 12p (specifically 12p11) is restricted to children aged 5 or over, nor to germinomatous tumours.

Furthermore, not all MGCTs in children 5 and over in our study followed the proposed type II cytogenetic profile. In comparison to the 14 MGCTs in children under 5, the 19 primary MGCTs in children of 5 or over showed minor increases in the frequency of gain on 2q, 3q, 12 and loss on 11q only, with none of these differences attaining even borderline statistical significance (data not shown). Moreover, loss on 11p or 18 was not seen in any of the 19 primary tumours of children aged 5 or more and gain of 12p was seen in only 10 cases (53%). It should be noted that 16 of the 20 MGCTs of children aged 5 and over were ovarian, cases that are under-represented in the published literature.

Our findings suggest that due to the relative lack of childhood MGCTs analysed to date, the proposed classification system may rely too heavily on data from adult TGCTs. This view would be concordant with known clinical outcome data, which indicates that childhood MGCTs can be treated differently to their adult counterparts without adverse outcomes well into adolescence (Mann *et al*, 2000). Where the biological cut-off between childhood and adult MGCTs occurs, and what biology underpins this, has still to be elucidated. However, any new classification of GCTs needs to take these issues into account and provide robust epidemiological, biological and therapeutically beneficial information. This should be possible by integrating data produced for adult and childhood MGCTs but needs to take account of transcriptional profiles, as well as the increasing volume of available genomic and epigenetic information.

## Figures and Tables

**Table 1:** Clinicopathological data for the 34 MGCTs of childhood analysed

**Sample**	**Histology**	**Histology subtype**	**Site**	**Age (years)**	**Gender**	**Stage**	**Outcome**	**EFS^a^ (months)**
1	Germinoma	Pure	Ovary	12	Female	1	Alive	47
2	Germinoma	Pure	Ovary	13	Female	3	Alive	46
3	Germinoma	Pure	Ovary	14	Female	2	Alive	40
4	Germinoma	Pure	Ovary	10	Female	1	Relapse	16
5	Germinoma	Pure	Ovary	6	Female	4	Alive	20
6	Germinoma	Pure	Ovary	12	Female	1	Dead	27
7	Germinoma	Pure	Ovary	12	Female	1	Alive	15
8	Germinoma	Pure	Ovary	13	Female	1	Relapse	23
9	Germinoma	Pure	Brain	16	Male	1	Alive	144
10	Germinoma	Pure	Brain	10	Female	1	Dead	14
11	Germinoma	Within teratoma	Ovary	12	Female	2	Alive	36
12	Embryonal carcinoma	Pure	Metastasis^b^	15	Male	4	Relapse	8
13	Yolk sac tumour	Pure	Testis	1	Male	1	Alive	51
14	Yolk sac tumour	Pure	Testis	1	Male	1	Alive	92
15	Yolk sac tumour	Pure	Testis	0	Male	1	Alive	37
16	Yolk sac tumour	Pure	Testis	4	Male	1	Alive	30
17	Yolk sac tumour	Pure	Testis	1	Male	1	Alive	14
18	Yolk sac tumour	Pure	Testis	0	Male	1	Alive	12
19	Yolk sac tumour	Pure	Testis	2	Male	1	Alive	44
20	Yolk sac tumour	Pure	Ovary	14	Female	4	Alive	78
21	Yolk sac tumour	Pure	Ovary	13	Female	2	Alive	58
22	Yolk sac tumour	Pure	Ovary	12	Female	1	Alive	28
23	Yolk sac tumour	Pure	Ovary	12	Female	1	Alive	69
24	Yolk sac tumour	Pure	Ovary^c^	9	Female	2	Alive	93
25	Yolk sac tumour	Pure	Ovary	14	Female	1	Relapse	2
26	Yolk sac tumour	Pure	Ovary	13	Female	3	Alive	72
27	Yolk sac tumour	Within teratoma	SCT	2	Female	4	Alive	55
28	Yolk sac tumour	Within teratoma	SCT	3	Female	4	Alive	21
29	Yolk sac tumour	Within teratoma	SCT	0	Female	4	Alive	42
30	Yolk sac tumour	Within teratoma	SCT	1	Male	4	Alive	28
31	Yolk sac tumour	Within teratoma	SCT	1	Male	1	Alive	54
32	Yolk sac tumour	Within teratoma	Brain	12	Male	1	Alive	136
33	Yolk sac tumour	Pure	Ovary	0	Female	2	Relapse	4
34	Yolk sac tumour	Pure	Vagina	1	Female	2	Alive	43

a EFS – Event-free survival.

b Pulmonary metastasis of a testicular primary.

c Contralateral streak ovary noted peri-operatively, SCT – sacrococcygeal teratoma.

**Table 2:** Summary data for 33 primary MGCTs (22 YSTs & 11 Germinomas), listing those CNIs that show a statistically significant association (*P*<0.05), with regard to a particular attribute. In the ‘Comparison’ column the numbers in brackets refer to the number of cases in the compared categories

**Comparison**	**Over-representation of CNI**	**CNI**	***P*-value**	**Difference (95%CI)**
Histology (germinoma *vs* YST) (11 *vs* 22)	YST	1p−	0.003	63.6% [27.0%, 88.1%]
		6q−	0.004	59.1% [21.9%, 82.9%]
		3p+	0.02	45.5% [7.1%, 72.5%]
Gonadal cases (ovarian *vs* testicular) (17 *vs* 7)	Testicular tumours	3p+	0.02	53.8% [8.3%, 91.8%]
		8q−	0.04	42.9% [0.2%, 84.8%]
Age (<5 *vs* ⩾5 years) (14 *vs* 19)	Age <5 years old	1p−	0.01	−43.6% [−76.2%, −9.1%]
		4q−	0.02	−41.4% [−73.6%, −6.3%]
		4p−	0.03	−37.6% [−71.3%, −2.6%]
		6q−	0.02	−43.2% [−75.8 %, −8.0%]
YST cases only, age (<5 *vs* ⩾5) (14 *vs* 8)	Age ⩾5 years old	3q+	0.03	50.0% [6.5%, 87.9%]
12p gain (association with other CNIs)	12p gain present (*n*=14)	12q+	0.01	46.6% [11.9%, 77.7%]
	12p gain absent (*n*=19)	16p−	0.04	−36.8% [−67.9%, −3.2%]
12p gain cases only, age (<5 *vs* ⩾5) (4 *vs* 10)	Age <5 years old	11q+	0.01	−75.0% [−99.8%, −15.9%]
		6q−	0.02	−70.0% [−98.3%, −9.2%]
		2p+	0.04	−65.0% [−99.4%, −3.8%]
		3p+	0.04	−65.0% [−99.4%, −3.8%]
YST cases only, 12p gain (association with other CNIs)	12p gain present (*n*=7)	6q−	0.009	60.0% [17.5%, 92.4%]
		12q+	0.01	57.1% [11.0%, 91.7%]
	12p gain absent (*n*=15)	16p−	0.04	−46.7% [−81.5%, −3.4%]

**Table 3:** Total number of paediatric (⩽16 years) MGCTs analysed by CGH reported in the literature to date, with relative frequencies of gain of proximal 12p (i.e. excluding isolated 12p13-pter) by age range

	**Age < 5 years**		**Age ⩾5 <16 years**	
	**Case mix**	**12p gain**	**Case mix**	**12p gain**
Korn *et al* (1996)	0	0	1 (1 YST)	1
Riopel *et al* (1998)	1 (1 mixed)	1	9 (6 DG, 2 Mixed, 1 YST)	8^d^
Kraggerud *et al* (2000)	0	0	3 (1 DG, 2 YST)	2
Mostert *et al* (2000)	5 (5 YST)	3^a^	0	0
Perlman *et al* (2000)	16 (16 YST)	1	0	0
Rickert *et al* (2000)	0	0	6 (3 Germinoma, 2 Mixed, 1 YST)	4
Schneider *et al* (2002)	9 (9 YST)	0	10 (4 Mixed, 3 YST, 2 CC, 1 EC)	6
Veltman *et al* (2005)	8 (8 YST)	1^b^	3 (1 DG, 1 YST, 1 Mixed)	2^e^
Schneider *et al* (2006)	1 (1 mixed)	0	9 (5 Mixed, 3 Germinoma, 1 YST)	7^f^
Present study	14 (14 YST)	3^c^	20 (9 DG, 8 YST, 2 Germinoma, 1 EC)	9^g^
				
Total	55	9 (16.4%)	61	39 (63.9%)

Case mix includes yolk sac tumour (YST), dysgerminoma (DG), choriocarcinoma (CC), embryonal carcinoma (EC) and mixed malignant germ cell tumour (mixed).

a 12p12–13 gain in all three cases.

b >5 MB gain, but incomplete gain of 12p without including 12p11 or 12p13-pter.

c Including one case showing whole chromosome gain (case 27), and a further case showing gain of 12p12-pter (case 34).

d High copy gain at 12p11 alone in two cases.

e Dual gain of 12p13-pter and 12p11 in one case.

f Including two cases showing whole chromosome gain (one with high copy gain of 12p), and one case of high copy gain at 12p12.

g Including one case showing gain of 12p12–13 (case 5), and a further case showing gain of 12p12-pter (case 2).

**Table 4:** Total number of paediatric (⩽16 years) MGCTs analysed by conventional cytogenetic techniques reported in the literature to date, with relative frequencies of iso(12p) by age range

	**Age < 5 years**		**Age ⩾5 <16 years**	
	**Case mix**	**iso(12p)**	**Case mix**	**iso(12p)**
Oosterhuis *et al* (1988)	1 (1 YST)	0	0	0
Speleman *et al* (1990)	0	0	1 (1 Mixed)	1
Shen *et al* (1990)	0	0	1 (1 Mixed)	0 (but 4 copies of chromosome 12)
Albrecht *et al* (1993)	0	0	1 (1 Germinoma)	0 (but extra 12p material)
de Bruin *et al* (1994)	0	0	1 (1 Mixed)	1
Perlman *et al* (1994)	6 (6 YST)	0	0	0
Stock *et al* (1994)	6 (2 EC, 2 YST, 2 Mixed)	1	5 (2 DG, 1 Mixed, 2 SE)	1
Jenderny *et al* (1995)	4 (4 YST)	4^a^	2 (2 YST)	1^a^
Stock *et al* (1995)	8 (6 YST, 2 EC)	1	5 (2 DG, 2 Mixed, 1 EC)	2
Sainati *et al* (1996)	1 (1 EC)	0	0	0
Lemos *et al* (1998)	1 (1 Geminoma)	0	0	0
Losi *et al*. (1994)	0	0	1 (1 Mixed)	1
Bussey *et al* (1999)	21 (11 YST, 7 Mixed, 3 UK)	0	23 (16 Mixed, 3 YST, 2 UK, 1 Germinoma, 1 Malignant teratoma)	2+2 other 12p structural abnormalities
Okada *et al* (2002)	0	0	10 (6 Germinoma, 3 Mixed, 1 YST)	2
Van Ecten *et al* (2002)	3 (3 YST)	0	0	0
Poulos *et al* (2006)	1 (1 Mixed)	1	2 (2 Mixed)	2
				
Total	52	7 (13.5%)	52	13 (25%)+4 others

Case mix includes mixed malignant germ cell tumour (mixed), yolk sac tumour (YST), embryonal carcinoma (EC), dysgerminoma (DG), seminoma (SE) and unknown germ cell tumour (UK).

a Gain of chromosome 12 by *in situ* hybridisation – iso(12p) status unclear, but one case in each age range almost certainly iso(12p).
